# Food antigens suppress small intestinal tumorigenesis

**DOI:** 10.3389/fimmu.2024.1373766

**Published:** 2024-09-18

**Authors:** Takaharu Sasaki, Yuna Ota, Yui Takikawa, Tommy Terrooatea, Takashi Kanaya, Masumi Takahashi, Naoko Taguchi-Atarashi, Naoko Tachibana, Haruka Yabukami, Charles D. Surh, Aki Minoda, Kwang Soon Kim, Hiroshi Ohno

**Affiliations:** ^1^ Laboratory for Intestinal Ecosystem, RIKEN Center for Integrative Medical Sciences, Yokohama, Japan; ^2^ Graduate School of Medical Life Science, Yokohama City University, Yokohama, Japan; ^3^ Laboratory for Cellular Epigenomics, RIKEN Center for Integrative Medical Sciences, Yokohama, Japan; ^4^ Department of Life Sciences, Pohang University of Science and Technology (POSTECH), Pohang, Republic of Korea; ^5^ Department of Cell Biology, Faculty of Science, Radboud Institute for Molecular Life Sciences, Radboud University, Nijmegen, Netherlands; ^6^ Laboratory for Immune Regulation, Graduate School of Medical and Pharmaceutical Sciences, Chiba University, Chiba, Japan

**Keywords:** food antigens, intestinal tumor, Peyer´s patches, M cells, microfold cells, scRNA-seq analysis, T cells, dendritic cells (DC)

## Abstract

Food components suppressing small intestinal tumorigenesis are not well-defined partly because of the rarity of this tumor type compared to colorectal tumors. Using *Apc^min/+^* mice, a mouse model for intestinal tumorigenesis, and antigen-free diet, we report here that food antigens serve this function in the small intestine. By depleting Peyer’s patches (PPs), immune inductive sites in the small intestine, we found that PPs have a role in the suppression of small intestinal tumors and are important for the induction of small intestinal T cells by food antigens. On the follicle-associated epithelium (FAE) of PPs, microfold (M) cells pass food antigens from lumen to the dendritic cells to induce T cells. Single-cell RNA-seq (scRNA-seq) analysis of immune cells in PPs revealed a significant impact of food antigens on the induction of the PP T cells and the antigen presentation capacity of dendritic cells. These data demonstrate the role of food antigens in the suppression of small intestinal tumorigenesis by PP-mediated immune cell induction.

## Introduction

Gastrointestinal tumors are a major health issue worldwide. While the number of deaths from gastrointestinal tumors is increasing, tumors of the small intestine are relatively rare, accounting for 3%–6% of all gastrointestinal neoplasms and 1%–3% of all gastrointestinal malignancies. However, neither non-invasive detection methods nor factors that are involved in the regulation of small intestinal tumors have been reported (1). Tumor diagnosis is often delayed until the late stages because of the asymptomatic features and, therefore, the identification of food components in our daily life that prevent/inhibit small intestinal tumorigenesis is important for reducing the disease incidence (2). Growing evidence does demonstrate that immune responses play important roles in the regulation of tumors in general.

In the gut, the immune system is continuously induced or regulated by factors such as food components and microbiota that constantly make contact with the gut. The immune system includes lymphocytes classified into adaptive immune cells and innate lymphoid cells (ILCs). Adaptive immune cells are further classified into CD4^+^ T cells including interferon-gamma (IFNγ)-producing type 1 T helper (Th1) cells, interleukin (IL)-4/IL-5/IL-13-producing type 2 T helper Th2 cells, IL-17/IL-22-producing type 17 T helper (Th17) cells, IL-10/transforming growth factor-beta (TGFβ)-producing regulatory T (Treg) cells, follicular helper T (Tfh) cells helping differentiation of B cells in the secondary lymphoid tissues, CD8^+^ T cells with cytotoxic functions, and B cells producing IgA (3). In the gut, γδ T cells and Eomes^+^CD4 T cells also contribute to the homeostasis of the mucosal immunity (4, 5). ILCs are classified into natural killer (NK) cells, ILC1, ILC2, and ILC3, functionally corresponding to CD8^+^ T cell, Th1, Th2, and Th17 cells, respectively, in terms of functions such as cytokine production, and exILC3, which is IFNγ-producing ILC3 (6, 7).

Peyer’s patches (PPs) are secondary lymphoid tissues located in the small intestine involved in the induction of intestinal immunity. Unlike other lymphoid tissues, PPs lack afferent lymphatics. Instead, they possess microfold cells (M cells), a specialized subset of epithelial cells uniquely adapted for the transepithelial transport of large luminal molecules or microbes, in the epithelial region covering PP lymphoid follicles called follicle-associated epithelium (FAE) (8). Within PPs, various myeloid cells are present, including dendritic cells (DCs) categorized into different subtypes: CD11c^-^CD8α^+^ conventional DC (cDC1), CD11c^+^CD8α^-^ conventional DC (cDC2), CD11c^-^CD8α^-^ conventional DC (DN-cDC2), lysozyme-expressing DC (LysoDC), plasmacytoid DC (pDC), and lysozyme-expressing macrophage (LysoMac) (9, 10). Functionally, cDC2, DN-cDC2, LysoDC, and LysoMac are considered to induce IgA production, cDC1 is for CD8^+^ T cell activation, and LysoDC is for Th1 cell activation (9, 10). In terms of tumor-regulating functions, gut microbiota induces immune cells such as interferon-gamma (IFNγ)-producing type 1 T helper (Th1) cells and CD8^+^ T cells, which provide a tumor-suppressive environment (11–14). Conversely, gut microbiota can also promote regulatory T-cell (Treg) differentiation that may aid tumor progression (15). Furthermore, the microbiota has been reported to have a role in regulating the pathogenesis of colorectal tumors and mediating tumor responses to chemotherapeutic agents and PD-1 immunotherapy (16–18). On the other hand, we have previously reported that food antigens have a great impact on the induction of immune cells in the small intestine rather than in the large intestine (19–21). Among T cells, food antigens primarily play a role in inducing small intestinal Th1 cells and Treg cells (19). Additionally, we have reported that food antigens promote IgA production by inducing follicular helper T cells, which, in turn, provide protection against *Salmonella enterica* serovar Typhimurium infection (21). Furthermore, other studies have demonstrated the potential contribution of food antigens to dampening type 2 immunity by inhibiting the proliferation and activation of type 2 innate lymphoid cells (ILC2s) (22). However, the role of food antigens in the regulation of intestinal tumors has not been examined. In the current study, we aimed to investigate the role of food antigens in the regulation of intestinal tumors using *Apc* ^min/+^ mice, which develop multiple tumors in both small and large intestines (23, 24). These mice carry a loss-of-function mutation in the tumor-suppression gene adenomatous polyposis coli (*Apc*), which, when mutated in the germline, is responsible for familial adenomatous polyposis (23, 24). We discovered that food antigens play a crucial role in suppressing small intestinal tumorigenesis. Here, we characterized the induction of the immune system by PPs in response to food antigens. These effects are suggested to be mediated by the induction of immune cells through PPs.

## Materials and methods

### Animal experiments

SPF and germ-free B6 mice were purchased from CLEA Japan. *Apc* ^min/+^ mice (Strain #002020) and *Villin*-Cre (Strain #004586) mice were purchased from Jackson Laboratories. *Spib* ^flox/flox^ mice (KOMP Repository) were crossed with *Villin*-Cre mice to obtain M-cell-deficient mice (25). Antigen-free diet (AFD) was produced as previously described (19–21). In the experiment using bovine serum albumin (BSA) or ovalbumin (OVA) as food antigens, they were dissolved in the AFD at 5% (w/v) concentration. The administration of the AFD or BSA/OVA-added AFD was begun at 4–5 weeks of age. *Apc* ^min/+^ mice were placed on the AFD under SPF conditions for 6 weeks for the analysis of tumors, and wild-type mice were maintained on the AFD under SPF or GF conditions for 4 to 5 weeks in order to analyze immune cells. After intestines were cut open longitudinally, the size and the number of tumors were measured under a stereomicroscope. PP-null wild-type (WT) mice were generated by intravenously injecting anti-IL-7Rα antibody (A7R34) into WT dams at E14.5, and their offspring were maintained on a normal chow diet (NCD) or an AFD for 5 weeks under SPF conditions beginning at 4–5 weeks of age. PP-null *Apc* ^min/+^ mice were generated in the same manner by injecting the A7R34 antibody (26, 27) into WT mice dams crossed with *Apc* ^min/+^ male mice at E14.5 and their *Apc* ^min/+^ offspring maintained on an NCD under SPF conditions were used for the analysis. In this study, we used both male and female mice in the *Apc* ^min/+^ mice experiment and the M-cell null mice experiment. In the experiment using PP-null mice, we used female mice. In these experiments, we obtained similar results (**Supplementary Figures 7**, **8**). We thus used male mice in the scRNA-seq experiment. Mice used in this study were on a C57BL/6 background and were maintained in the RIKEN Center for Integrative Medical Sciences under specific pathogen-free (SPF) conditions or germ-free conditions in vinyl isolators at RIKEN or Yokohama City University. All animal experiments were conducted in accordance with the guidelines of the Institutional Animal Care and Use Committee of RIKEN or Yokohama City University.

### Flow cytometry

The following are antibodies used for FACS staining: anti-CD3ε (clone 145-2C11; FITC and BV421), anti-CD4 (clone GK1.5; APC, PE/Cy7 and Biotin), anti-CD8α (clone 53-6.7, PerCP/Cy5.5 and Biotin), anti-B220 (clone RA3-6B2; FITC and Biotin), anti-CD19 (clone 1D3; PE), anti-NK1.1 (clone PK136; APC/Cy7), anti-IgA (clone mA-6E1, PE) anti-CD45.2 (clone 104; PerCP/Cy5.5 and Biotin), anti-CD62L (clone MEL-14; FITC), anti-CD44 (clone IM7, APC/Cy7), anti-CD11b (clone M1/70; PE/Cy7), anti-CD11c (clone N418; PE) anti-I-A/I-E (M5/114.15.2; BV421), anti-PD-1 (clone 29F.1A12, BV711), anti-CXCR5 (clone L138D7; Biotin), and anti-T-bet (clone 4B11; PE/Cy7) antibodies were purchased from BioLegend. APC Streptavidin, APC/Cy7 Streptavidin, and BV605 Streptavidin were also purchased from BioLegend. Anti-CD16/32 (clone 2.4G2; purified) and anti-Rorγt (clone Q31-378; BV421) antibodies were purchased from BD Biosciences. Anti-Foxp3 (clone FJK-16s; PE) antibody was purchased from Thermo Fisher Scientific.

Prepared cell suspensions were stained with Zombie Aqua Fixable Viability Kit (BioLegend) to stain dead cells and treated with anti-CD16/32 (2.4G2) antibody for FcR-blocking. After washing, samples were stained with the following antibodies and analyzed using a BD FACSAria III or FACSCantoII (BD Biosciences). For detecting small intestinal lamina propria (SI LP) T cells and large intestinal lamina propria (LI LP) T cells, prepared cell suspensions were stained with antibodies for CD3ε (145-2C11), CD4 (GK1.5), CD8α (53-6.7), NK1.1 (PK136), and CD45.2 (104) followed by intracellular staining with antibodies for T-bet (4B11), Rorγt (Q31-378), and Foxp3 (FJK-16s) using the eBioscience™ Foxp3/Transcription Factor Staining Buffer Set (Thermo Fisher Scientific). Th1 cells, Treg cells, and CD8^+^ T cells were gated as CD3ε^+^NK1.1^-^CD4^+^T-bet^+^Rorγt^-^, CD3ε^+^NK1.1^-^CD4^+^Foxp3^+^, and CD3ε^+^NK1.1^-^CD8α^+^ cells, respectively. For detecting SI LP IgA-positive plasma cells, cell suspensions were stained with antibodies for CD19 (1D3), B220 (RA3-6B2), and IgA (mA-6E1), and IgA-positive plasma cells were gated as CD19^+^B220^-^IgA^+^ cells. For staining PP T cells, cell suspensions were staining with antibodies against CD3ε (145-2C11), CD19 (RA3-6B2), CD4 (GK1.5), CD8α (53-6.7), CD62L (MEL-14), CD44 (IM7), PD-1 (29F.1A12), and CXCR5 (L138D7). Among CD3ε^+^CD4^+^ T cells and CD3ε^+^CD8α^+^ T cells, naïve T cells and effector T cells were detected as CD62L^+^CD44^low^ and CD62L^-^CD44^high^ populations, respectively. Tfh cells were detected as CD3ε^+^CD4^+^PD-1^+^CXCR5^+^ cells. For the detection of PP DCs, cell suspensions were stained with antibodies against CD3ε (145-2C11) , B220 (RA3-6B2), CD11c (N418), I-A/I-E (M5/114.15.2), CD11b (M1/70), CD8α (53-6.7), and CD45.2 (104), and DCs were detected as CD45.2^+^CD3ε^-^B220^-^CD11c^+^I-A/I-E^+^ cells and they were further subdivided by the expression levels of CD11b and CD8α.

### Hybridoma culture for anti-IL-7Rα (clone A7R34) antibody purification

The hybridoma was cultured in the cell compartment of CELLine1000 (Integra Biosciences) starting from 2.5 × 10^7^ cells in 25 mL of RPMI 1640 medium containing 10% (v/v) FCS, MEM Non-Essential Amino Acids Solution (Gibco), 10 mM HEPES (Gibco), 1 mM sodium pyruvate (Sigma), 100 U/mL penicillin–streptomycin, 2 mM L-glutamine, and 0.1% (v/v) 2-mercaptoethanol (Thermo Fisher). The medium compartment was filled with 500 mL of the same medium without FCS and replaced with fresh medium twice per week. At the same time, the cell compartment was recovered and centrifuged at 4°C and 500*g* for 5 min to collect the supernatant for antibody purification and one-fifth of the hybridoma was suspended into 25 mL of media and returned into the cell compartment. Using the collected supernatant, ammonium sulfate (Nacalai Tesque) precipitation was carried out by adding the salt to 65% (w/v). The subfractioned protein suspension was centrifuged at 4°C and 15,000*g* for 60 min. After discarding the supernatant, the pellet was resuspended into PBS and put into clamped dialysis-tube Spectra/Por 1 (6–8 kD, REPLIGEN), which was prepared by boiling in distilled water for 20 min. Dialysis was performed by placing the tubes in 2 L of PBS pre-cooled at 4°C and the PBS was exchanged at 1, 2, 3, 6, 26, 36, and 50 h. The dialyzed solution was filtered through a 0.22-μm filter, and the antibody was purified by HiTrap Protein G HP (Merck). Elution was performed with 0.1 M glycine-HCl, pH 2.7, and collected into 1 M Tris-HCl (pH 8.0). The eluted antibody was concentrated with an Amicon Ultra-15 Centrifugal Filter Unit (Merck), filtered through a 0.22-μm filter, and stored at a concentration of 0.5 mg/mL in PBS.

### Ligated Peyer’s patch intestinal loop assay

The intestinal loop assay was performed as described previously (28). Briefly, mice housed on NCD under SPF conditions were fasted for 5 h and anesthetized by i.p. injection of a cocktail consisting of 3% (v/v) medetomidine, 8% (v/v) midazolam, and 10% (v/v) butorphanol mixed in deionized distilled water (100 μL/g body weight). Both ends of the distal part of the small intestine containing three or four PPs were ligated by sutures. The ligated part of the intestine was injected with 5 or 20 mg of FITC-conjugated OVA (FITC-OVA). After 1 h, mice were sacrificed and PPs were collected for further analysis.

FITC-OVA was prepared by mixing OVA and FITC-I (Dojindo). OVA in PBS was added at a ratio of 1:100 FITC-I in 0.5 M carbonate buffer and incubated for 1 h at room temperature. To remove unreacted FITC-I, the mixture was filtered through and concentrated by an Amicon ultra-15 50K device (Merck Millipore). A Pierce BCA protein assay kit (Thermo) was used for measuring the concentration according to the manufacturer’s protocol.

### Cell preparation from tissues

To prepare cells from the SI LP, after removing mesenteric adipose tissue and PPs with scissors, the small intestine was cut open longitudinally and the intestinal contents were washed out with PBS. The small intestine was cut into 1-cm pieces and stirred in a 100-mL conical flask containing 40 mL of 1 mM EDTA in PBS with a stir bar at 37°C and 500 rpm for 20 min. After the pieces were shaken vigorously in 20 mL OF PBS, they were minced into 1- to 2-mm pieces with scissors and stirred in a 20-mL conical flask containing 10 mL of RPMI medium containing 2% (v/v) FCS, 1 mg/mL collagenase (WAKO), and 50 μg/mL DNase (“collagenase-medium”) with a stir bar at 37°C and 500 rpm for 15 min. After allowing the contents of the flask to settle for 1 min to let the remaining tissue pieces sink to the bottom, 7 mL of the top clear medium was collected and filtered through a 100-μm cell strainer (BD Falcon). The remaining tissue suspension was passed through an 18-G needle 10 times, 7 mL of collagenase medium was added to the flask, and the sample was stirred again at 37°C and 500 rpm for an additional 5 min. Samples were filtered through a 100-μm cell strainer and combined with the medium previously collected from the same sample. After centrifugation at 4°C and 500*g* for 5 min, pellets were resuspended in 10 mL of 40% Percoll PLUS (GE Healthcare) and 2 mL of 75% Percoll PLUS was layered under the cell suspension. The tubes were centrifuged at room temperature and 500*g* for 20 min with the brake off, and the cells were collected from the interface of the Percoll gradient into 10 mL of 5% FCS-Hanks’ Buffer. Samples were centrifuged at 4°C and 500*g* for 5 min and then used for subsequent analysis. SI LP cells or LI LP cells of *Apc* ^min/+^ mice were prepared from the tissues cut off the tumors visible under a stereomicroscope by micro scissors.

Cells in the LI LP were prepared using the same protocol as for the preparation of SI cells except that the concentration of EDTA was 5 mM and that the minced tissue was stirred in the collagenase medium for 30 min both before and after passage through the needle.

For PPs and tumors dissected from *Apc* ^min/+^ mice, tissues were minced with scissors and then stirred in a 20-mL conical flask containing 10 mL of collagenase medium with a stir bar at 37°C and 500 rpm for 15 min. The suspension was passed through an 18-G needle 10 times and stirred at 37°C and 500 rpm for an additional 15 min. Samples were filtered through a 100-μm cell strainer, centrifuged at 4°C and 500*g* for 5 min, and then used for analysis.

### Isolation and whole-mount immunostaining of FAE

PPs dissected from mice were incubated with 30 mM EDTA/HBSS for 20 min on ice, and then FAE was peeled off from PPs by syringe needles under the stereomicroscope observation. For the whole-mount immunostaining, isolated FAE was transferred to a glass-bottom chamber and fixed with 4% paraformaldehyde (Nacalai Tesque) for 1 h on ice. Fixed FAE specimens were permeabilized with 0.1% (v/v) Triton X-100 in PBS and were incubated with purified rat anti-GP2 (clone 2F11-3 generated in our laboratory) overnight at 4°C. Subsequently, the specimens were incubated with donkey anti-rat IgG conjugated to Cy3, Alexa 647 phalloidin, and DAPI for 2 h at room temperature. The images were captured by an SP8 confocal laser scanning microscope (Leica).

### Single-cell RNA-sequencing analysis

The library was prepared using Chromium Single Cell 3’ Library & Gel Bead Kit v2 (10x Genomics). Sequencing was performed by Takara Bio using NovaSeq 6000 (Illumina) with a NovaSeq 6000 S4 Reagent Kit (Illumina) and a NovaSeq Xp 4-Lane Kit (Illumina). FASTQ raw files were processed by Cell Ranger v3.0.2 software and aligned to the mouse reference genome mm10. Files generated by Cell Ranger were imported into R (ver 4.1.2) and processed by Seurat (ver 3.1.5) according to the tutorials provided by the Setija Lab (https://satijalab.org/seurat/index.html) (29). For the analysis of lymphocytes and myeloid cells, targeted clusters were extracted by the “subset” function, followed by performing linear dimensional reduction, clustering, and running non-linear dimensional reduction by UMAP. Seurat objects derived from T cells and DC analysis were merged again, and ligands expressed by DCs acting on naïve T cells were detected by NichNet (30). In these experiments, the cell numbers of each population were calculated by multiplying the total cell numbers of PPs, the percentage of sorted cells, and the proportions of each detected population in the scRNA-seq analysis.

## Results

**Figure 1:**
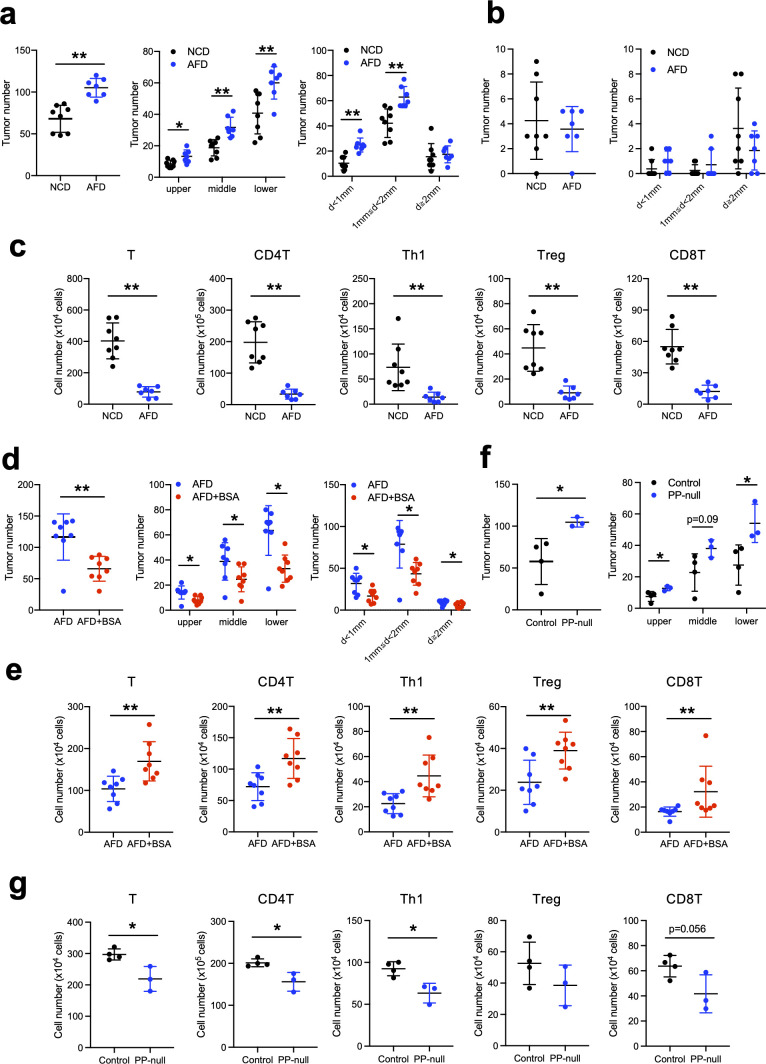
Food antigens suppress small intestinal tumorigenesis. **(A–C)** *Apc* ^min/+^ mice were placed on a normal chow diet (NCD) or an antigen-free diet (AFD) for 6 weeks starting at 4 to 5 weeks of age (NCD; *n* = 8, AFD; *n* = 7). The number of total small intestinal tumors and the numbers and sizes of the tumors in the small intestine equally divided into three parts from the proximal to the distal end (upper, middle, and lower) were measured **(A)**. The numbers and sizes of the colonic tumors **(B)**. Numbers of small intestinal lamina propria (SI LP) T cells, CD4^+^ T cells, Th1 cells, Treg cells, and CD8^+^ T cells were determined by flow cytometry analysis **(C)**. **(D, E)** *Apc* ^min/+^ mice were placed on the AFD or AFD supplemented with 5% BSA for 6 weeks starting at 4 to 5 weeks of age (*n* = 8). The numbers and sizes of the small intestinal tumors **(D)** and cell numbers of SI LP T cells were determined by flow cytometry analysis **(E)**. **(F, G)** PP-null *Apc* ^min/+^ mice were obtained as described in Materials and Methods. The number of tumors in the small intestine from control and PP-null mice was measured **(F)** and the numbers of SI LP T cells were calculated by flow cytometry analysis **(G)** (*n* = 3–4). Data are presented as mean ± SD. Statistical significance was calculated by Mann–Whitney *U*-test **(A–E)** and Student’s *t*-test **(F, G)**. *p < 0.05 and **p < 0.01.

To clarify the role of food antigens in the regulation of intestinal tumorigenesis, we made use of *Apc* ^min/+^ mice. The *Apc* ^min/+^ mice were maintained on a laboratory-made AFD lacking antigenic molecules larger than 10 kDa (19–21) for 5–6 weeks. Strikingly, the number of small intestinal tumors was significantly increased in AFD-fed mice compared to NCD-fed mice (**Figure 1A**). When the small intestine was equally divided into three parts, the number of tumors was increased in all three regions (**Figure 1A**). The number of tumors smaller than 2 mm in diameter was increased with the AFD compared to the NCD, despite there being similar numbers of tumors 2 mm or larger, presumably indicating that the tumorigenesis process had been affected by the AFD (**Figure 1A**). On the other hand, the number of colonic tumors was comparable between the AFD- and NCD-fed mice (**Figure 1B**). Because of the association of immune cells with intestinal tumorigenesis (31), we also enumerated the small intestinal lamina propria (SI LP) T cells and observed a marked decrease of SI LP T cells in *Apc* ^min/+^ AFD- compared to NCD-fed mice (**Figure 1C**), suggesting the possibility that induction of immune cells in the small intestine suppresses tumorigenesis. The AFD had been previously used to examine the role of food antigens in the induction of small intestinal immune cells (19–21); however, it is possible that differences in the composition of ingredients between the AFD and NCD resulted in the increased number of tumors in AFD-fed mice. In order to further dissect the role of food antigens in the regulation of tumorigenesis, BSA was dissolved in the AFD as a model food antigen and *Apc* ^min/+^ mice were placed on this diet for 6 weeks. The amount of BSA was calculated so that the amount would be equivalent or less than the total proteins contained in the NCD. As expected, addition of BSA decreased the number of small intestinal tumors to levels similar to the NCD-fed mice (**Figures 1A, D**). Moreover, the number of SI LP T cells was restored by the addition of BSA (**Figure 1E**). These data suggest that food antigens suppress small intestinal tumorigenesis through the induction of T cells, but not likely through the differences in nutritional composition between NCD and AFD.

In order to examine the causal relationship between the induction of T cells and the suppression of tumorigenesis, we examined the role of PPs in the suppression of tumorigenesis. For this purpose, we generated PP-null *Apc* ^min/+^ mice by intravenously injecting anti-IL-7Rα antibody (A7R34) into *Apc* ^min/+^ dams at E14.5 and then used their offspring for the analysis (26). PP-null *Apc* ^min/+^ mice on an NCD had an increased number of tumors, comparable to that observed in AFD-fed *Apc* ^min/+^ mice, in all parts of the small intestine, and this was associated with decreased SI LP T cells (**Figures 1A, F, G**). Specifically, the number of IFNγ-producing Th1 cells was significantly decreased. Physiological production of IFNγ has also been reported to suppress tumorigenesis in *Apc* ^min/+^ mice (32). Taken together, these data indicate that the induction of SI LP T cells, especially Th1 cells, through PPs has a role in the suppression of small intestinal tumorigenesis.

**Figure 2:**
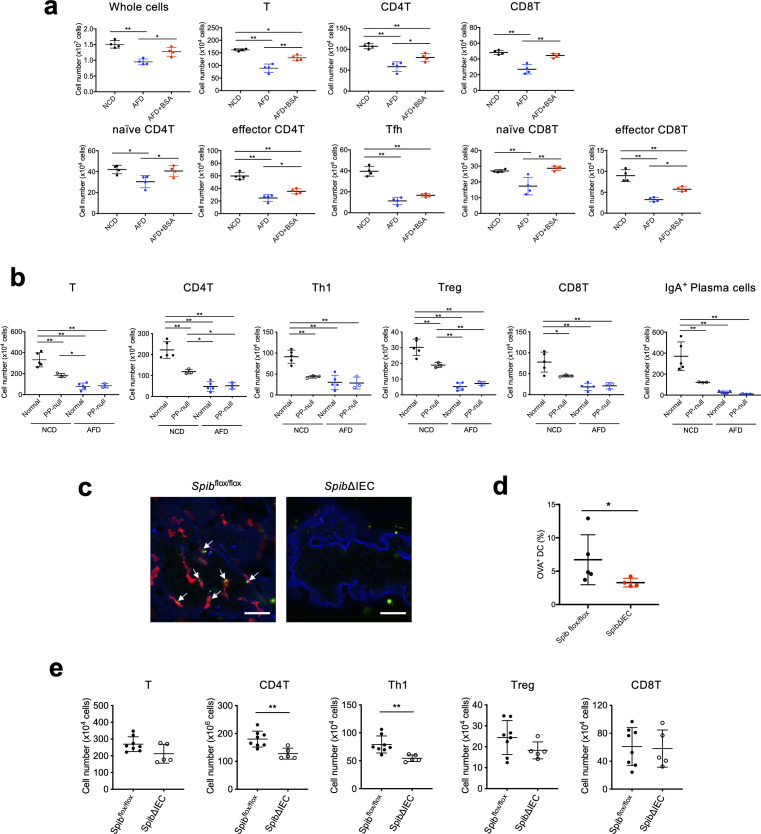
Peyer’s patches are involved in the induction of SI LP T cells by food antigens and the suppression of small intestinal tumorigenesis. **(A)** WT mice were placed on an NCD, AFD, or 5%BSA-AFD for 5 weeks under SPF conditions starting at 4 weeks of age, and the numbers of PP T cells, CD4^+^ T cells, and CD8^+^ T cells, including naive cells, effector cells, and Tfh cells, in PPs were determined by flow cytometry analysis (*n* = 4). **(B)** PP-null mice and control mice were obtained as described in Materials and Methods. They were placed on an NCD or AFD starting at 4 weeks of age for 5 weeks under SPF conditions, and the number of the SI LP T cells and IgA^+^ plasma cells were determined by flow cytometry analysis (*n* = 3–5). **(C)** *Villin* ^+/+^ *Spib* ^flox/flox^ and *Villin* ^Cre/+^ *Spib* ^flox/flox^ (*Spib*ΔIEC) mice maintained on an NCD under SPF conditions were fasted for 5 h followed by anesthesia and the ligated small intestinal loops, including PPs, were injected with FITC-conjugated OVA dissolved in 1 mL of PBS. One hour after the injection, the FAE isolated from the extracted PPs was immunostained with anti-GP2 antibody, and samples were counterstained with fluorescent-labeled phalloidin. GP2-positive microfold cells are in blue, F-actin is in blue, and FITC-OVA is in green. The scale bars represent 20 μm. **(D)** One hour after the injection of FITC-conjugated OVA using the same loop assay methods as described above, PPs from *Villin* ^+/+^ *Spib* ^flox/flox^ and *Villin* ^Cre/+^ *Spib* ^flox/flox^ mice maintained on an NCD under SPF conditions were extracted and the cell suspensions were prepared by enzymatic digestion. Percentages of OVA-positive dendritic cells were measured by flow cytometry analysis (*n* = 4–5). **(E)** The numbers of SI LP T cells, CD4^+^ T cells, Th1 cells, Treg cells, and CD8^+^ T cells of *Villin* ^+/+^ *Spib* ^flox/flox^ and *Villin* ^Cre/+^ *Spib* ^flox/flox^ (*Spib*ΔIEC) mice maintained on an NCD under SPF conditions were determined by flow cytometry analysis (*n* = 4–5). Data are presented as mean ± SD. Statistical significance was calculated by Tukey–Kramer test **(A, B)** and Mann–Whitney *U*-test **(D, E)**. *p < 0.05 and **p < 0.01.

Given that PPs are significantly involved in the induction of SI LP T cells and the suppression of tumorigenesis by food antigens, we next used wild-type (WT) SPF mice to investigate whether PP lymphocytes themselves are also regulated by food antigens. The numbers of both CD4 and CD8 PP T cells, including naïve and effector T cells, were decreased in WT mice on an AFD and were restored by the addition of BSA to the AFD (**Figure 2A**), indicating that PP T cells were induced by food antigen. Because gut microbiota has also been reported to induce T cells in the intestine, we examined the number of PP T cells in a similar experiment using germ-free (GF) WT mice and obtained results similar to the SPF WT mice (**Supplementary Figure 1A**). These data suggest that food antigens have the potential to induce PP T cells independently of microbiota. Addition of OVA into the AFD as a different food antigen also restored the numbers of PP T cells, suggesting that the effect is not antigen-specific (**Supplementary Figure 1B**). In both SPF and GF conditions, the number of PP Tfh cells as well as other T cells was decreased in AFD-fed mice; however, Tfh cells were not restored by the addition of BSA into the AFD (**Figure 2A**, **Supplementary Figure 1A**). Accordingly, the decrease in the number of SI LP IgA^+^ plasma cells in AFD-fed mice was also not restored by the addition of BSA (**Supplementary Figure 2A**). SI LP T cells were induced by food antigen in GF mice as in SPF mice (**Supplementary Figures 2A, B**). In contrast, the number of large intestinal lamina propria (LI LP) T cells was not affected by food antigens in SPF mice (**Supplementary Figure 2C**). Overall, the present study indicates that food antigens specifically contribute to the induction of T cells in the small intestine, as we have previously shown (19).

We further evaluated the importance of PPs in the induction of SI LP T cells by food antigens. To this end, the anti-IL-7Rα antibody (A7R34) was intravenously injected into WT dam mice at E14.5 (26) and the resultant PP-null offspring were placed on an NCD or AFD. The PP-null mice fed with the food antigen-containing NCD showed significantly decreased numbers of SI LP T cells, including Th1 cells, Treg cells, and CD8^+^ T cells, as well as SI LP IgA^+^ plasma cells (**Figure 2B**), indicating the importance of PPs in the induction of SI LP immune cells. Of note, the AFD-fed mice were almost devoid of these SI LP T cells, even in PP-sufficient normal control mice (**Figure 2B**), indicating the importance of food antigens in the induction of SI LP T cells. The observed PP-independent SI LP T-cell induction could be attributable to the intact development of other gut-associated lymphoid tissue (GALT) structures such as ILF (33). There might also be a possible contribution of goblet cells or antigen-presenting cells outside of the PPs in the intestine (34–36).

The requirement for PPs in the induction of SI LP T cells by food antigens raises the possibility that PPs are the site where food antigens are taken up from the gut lumen. Therefore, we examined if M cells are involved in taking up food antigens. For that purpose, ligated intestinal loops containing a PP in *Villin* ^+/+^ *Spib* ^flox/flox^ mice and M-cell-deficient *Villin* ^Cre/+^ *Spib* ^flox/flox^ mice (37) were injected with FITC-conjugated OVA and the localization of OVA was determined by whole-mount immunohistochemistry with the M-cell marker GP2 (28, 38). Confocal imaging showed that OVA was present on the FAE and mainly co-localized with GP2, indicating that food antigens are taken up from the gut lumen into M cells (**Figure 2C**). In order to further examine the role of M cells in passing food antigens to antigen-presenting DCs in PPs, we performed the ligated loop assay in M-cell-deficient *Villin*-Cre *Spib* ^flox/flox^ mice and evaluated the percentages of OVA-positive PP DCs. Flow cytometric analysis showed that OVA^+^CD3ε^-^B220^-^CD11c^+^MHCII^+^ PP DCs were decreased in the M-cell-deficient mice, indicating that food antigens are mainly taken up through M cells into PPs and then passed to DCs for the induction of immune cells (**Figure 2D**). To further examine the role of M cells in the induction of SI LP T cells by food antigens, we analyzed the numbers of SI LP T cells in M-cell-deficient *Villin*-Cre *Spib* ^flox/flox^ mice. The number of SI LP Th1 cells in these mice was decreased compared to that of control mice, suggesting that food antigens passed through M cells are involved in the induction of SI LP Th1 cells (**Figure 2E**). In contrast to the findings in PP-null mice, there was no significant decrease in the number of the other SI LP T cell subsets in NCD-fed *Villin*-Cre *Spib* ^flox/flox^ mice (**Figures 2B, E**). This could presumably be due to a large contribution of the cells induced by food antigen in SI for the SI LP Th1 cells (19), whereas the other SI LP T cells in M-cell-deficient mice could be replenished by a homeostatic proliferation of T cells induced in lymphoid tissues other than PP. There might also be a possible contribution of other entry pathways for the food antigens into PPs, which then provide suitable environments for the induction of other types of T cells. Indeed, the presence of M-cell-independent mechanisms for protein transport through the FAE into PPs has been reported (39). Interestingly, we observed a trend toward increased numbers of the small intestinal tumors in M-cell-deficient *Apc* ^min/+^ mice, suggesting that M cells are involved in the suppression of the small intestinal tumorigenesis (**Supplementary Figure 3**).

**Figure 3:**
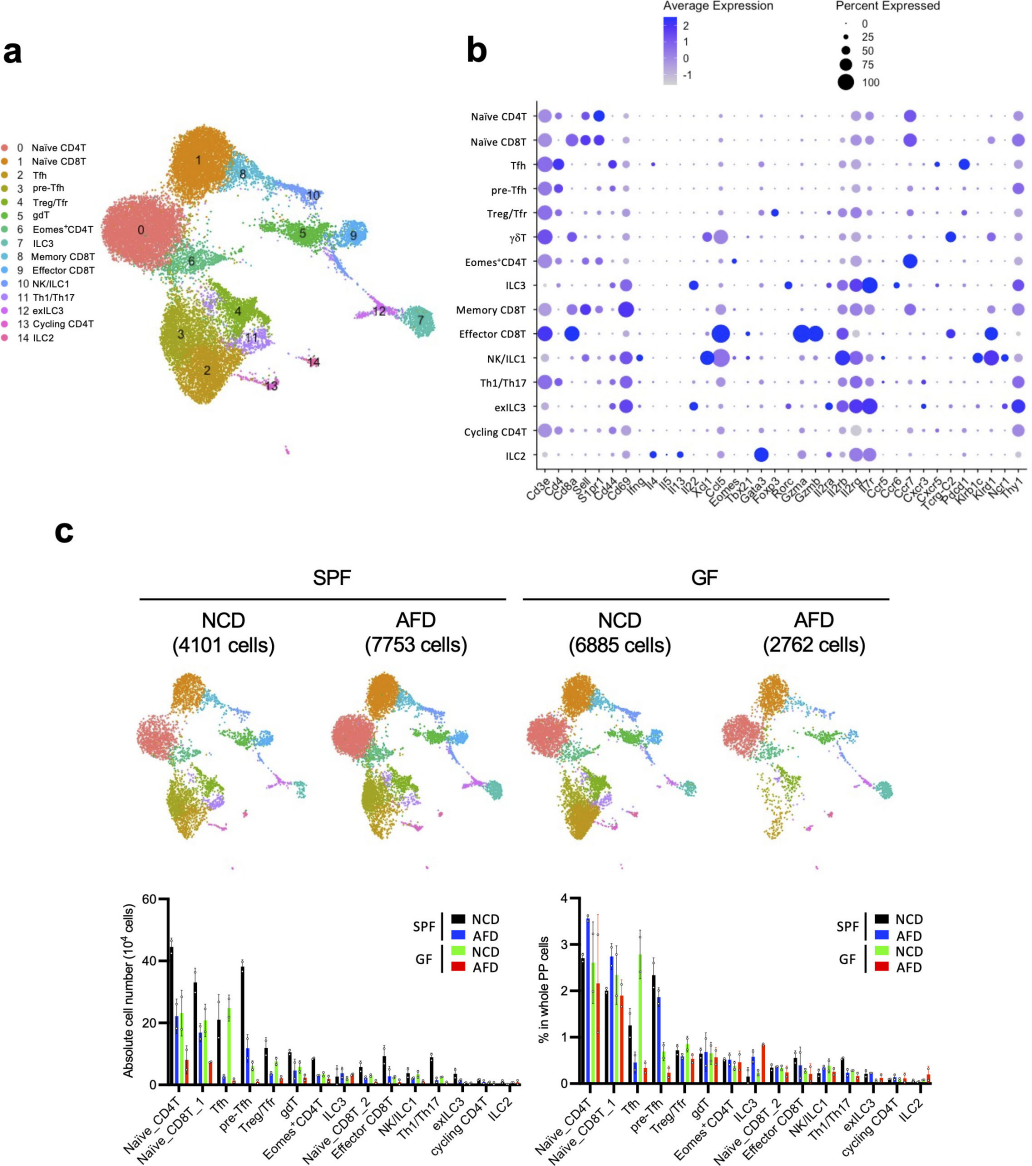
Comprehensive survey of the impact of food antigens on the induction of PP T cells. **(A)** WT mice kept under SPF or GF conditions were fed an NCD or AFD for 5 weeks starting at 4 weeks of age and CD19^-^ cells were depleted by FACS from enzymatically digested PP cells for single-cell RNA-seq analysis. Detected lymphocytes are illustrated in UMAP. **(B)** Average expression levels of the marker genes and percent of the expressed cells are illustrated as a DotPlot for each population shown in **(A)**. **(C)** UMAP of PP lymphocytes in SPF and GF mice maintained on an NCD or AFD and absolute cell numbers/percentages in whole PP cells for each cluster among these conditions (*n* = 2). Data are presented as mean ± SD. [**(B)**, UMAP in **(A, C)**] Data obtained from two mice were combined.

Because of the significant contribution of food antigens to the induction of PP T cells, we further examined the comprehensive landscape of these cells and the role of food antigens in their induction by single-cell RNA-seq (scRNA-seq) analysis. Since the majority of PP lymphocytes are CD19-positive B cells, CD19-negative cells were sorted from NCD- or AFD-treated mice housed under both SPF and GF conditions in order to enrich for T cells and were analyzed with a 10x Genomics platform. This analysis resulted in the classification of lymphocytes into 15 clusters including T cells and ILCs as shown by UMAP (**Figure 3A**). They were annotated according to the expression of marker genes characteristic of each population, including cell surface markers/receptors (*Cd3e*, *Cd4*, *Cd8a*, *Sell*, *S1pr1*, *Cd44*, *Cd69*, *Tcrg-C2*, *Pdcd1*, *Klrb1c*, *Klrd1*, *Ncr1*, and *Thy1*), cytokines and chemokines (*Ifng*, *Il4*, *Il5*, *Il13*, *Il22*, *Xcl1*, and *Ccl5*), transcription factors (*Eomes*, *Tbx21*, *Gata3*, *Foxp3*, and *Rorc*) and cytokine/chemokine receptors (*Il2ra*, *Il2rb*, *Il2rg*, *Il7r*, *Ccr5*, *Ccr6*, *Ccr7*, *Cxcr3*, and *Cxcr5*) (**Figure 3B**, **Supplementary Figure 4**). As a result, T cells were clustered into naïve CD4^+^ T, Tfh, pre-Tfh, Treg/Tfr, Eomes^+^CD4^+^ T, Th1/2/17, cycling CD4^+^ T, naïve CD8^+^ T cells, memory CD8^+^ T, effector CD8^+^ T, and γδ T cells, and ILCs were clustered into NK/ILC1, ILC2, ILC3, and exILC3 (**Figures 3A, B**). T cells were decreased in AFD-fed mice under both SPF and GF conditions, confirming that food antigens significantly contribute to the induction of T-cell subsets detected in this analysis (**Figure 3C**). By contrast, food antigens did not noticeably affect the numbers of ILC2, ILC3, and exILC3, although NK/ILC1 cells were decreased in AFD-fed mice (**Figure 3C**).

**Figure 4:**
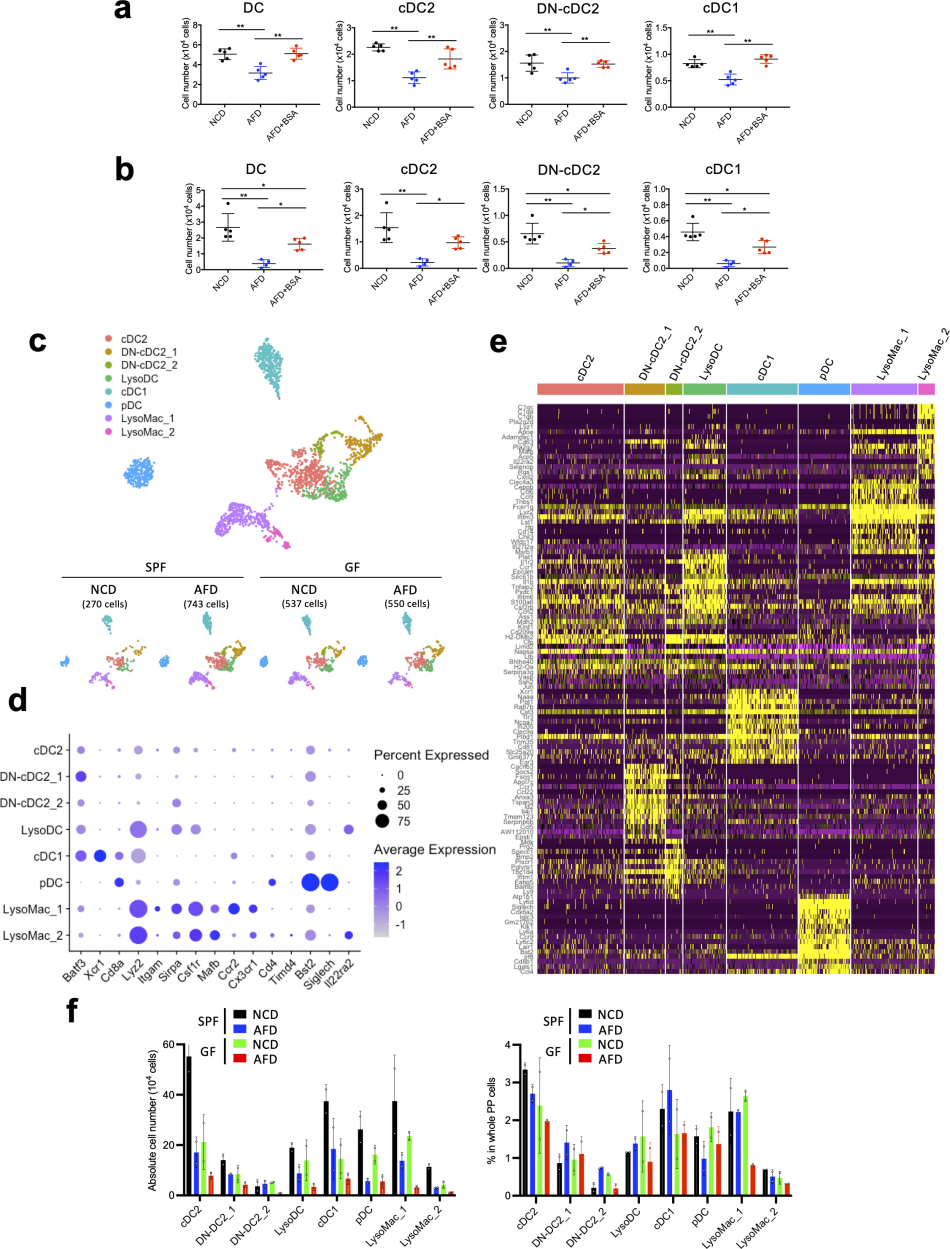
Effect of food antigens on DCs in PPs. **(A, B)** WT mice housed under SPF **(A)** or GF **(B)** conditions were placed on an NCD, AFD, or AFD supplemented with 5% BSA for 4 weeks starting at 4 weeks of age and the numbers of PP DCs, cDC2, DN-cDC2, and cDC1 were determined by flow cytometry analysis (*n* = 4–5). **(C)** Enzymatically digested and sort-purified CD19^-^ PP cells from NCD- and AFD-fed mice under SPF or GF conditions were analyzed by single-cell RNA-seq analysis. Detected myeloid cell clusters are illustrated in UMAP. **(D)** Average expression levels of the marker genes and percent of the expressed cells are illustrated as a dot plot for each myeloid cell cluster. **(E)** Expression of the top 15 marker genes for each myeloid cell cluster depicted in **(C)** is illustrated as a heatmap. **(F)** Absolute cell numbers and percentages in whole PP cells of each myeloid cell cluster in NCD- or AFD-fed mice kept under SPF or GF conditions (*n* = 2). **(C–E)** Data obtained from two mice were combined. Data are presented as mean ± SD. **(A, B)** Statistical significance was calculated by Tukey–Kramer test. *p < 0.05 and **p < 0.01.

**Figure 5:**
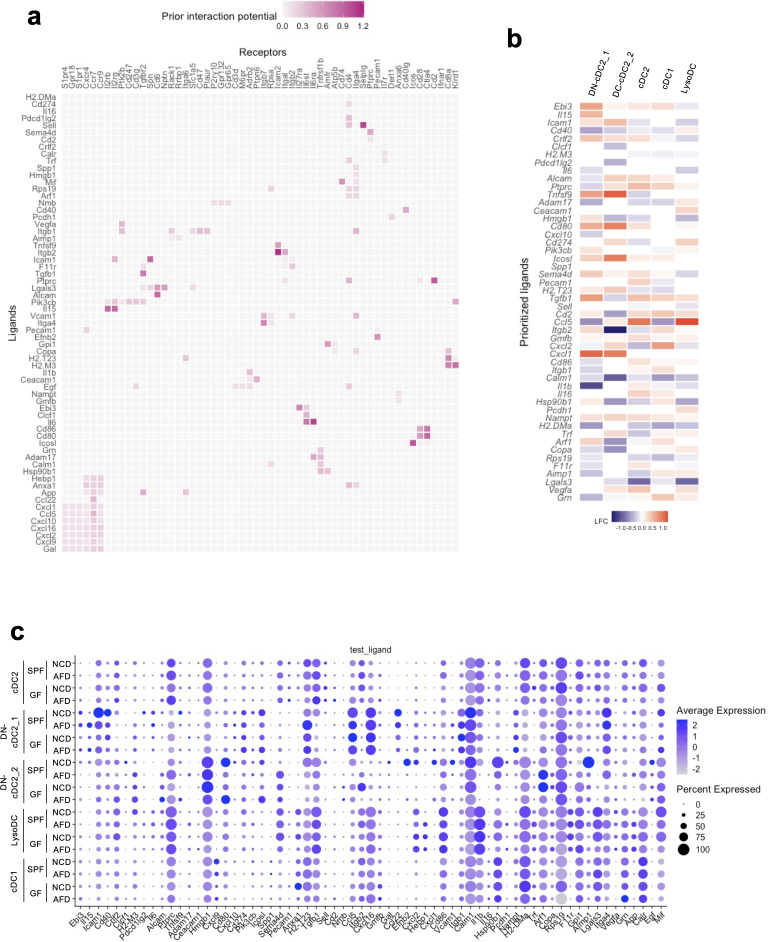
Prediction of ligand–receptor interactions between DCs and naïve T cells causing functional changes in T cells induced by food antigens. **(A–C)** Single-cell RNA-seq analysis data were analyzed by NicheNet. **(A)** Predicted interactions between ligands expressed by DCs and receptors expressed by naïve T cells causing gene expression changes in naïve T cells by food antigens. **(B)** Putative ligands expressed by each DC subset changed by the absence of food antigens that potentially could act on the corresponding receptors expressed by naïve T cells. The heatmap shows the log2 fold change (LFC) of the gene expression in GF mice maintained on an AFD compared to those on an NCD. **(C)** Average expression and percentage of cells expressing the ligand detected by NicheNet within the indicated DC subsets in SPF and GF mice maintained on an NCD or AFD. **(A–C)** Data obtained from two mice were combined.

Given that DCs receive food antigens from the gut lumen (**Figure 2D**), we further analyzed the role of food antigens in the regulation of PP DCs, which are classically divided into three subsets: CD11b^+^CD8α^-^ cDC2, CD11b^-^CD8α^-^ double-negative (DN)-cDC2, and CD11b^-^CD8α^+^ cDC1 (40–42). Flow cytometric analysis revealed that numbers of these DCs were reduced in AFD-fed mice and were restored by BSA addition under SPF conditions (**Figure 4A**). Similar results were obtained under GF conditions as well, indicating that DCs are regulated by food antigens (**Figure 4B**). Because of the reported heterogeneity of the constituents of PP mononuclear phagocytes, we further analyzed the detailed effects of food antigens on their cellularity by scRNA-seq analysis (10). They were classified into cDC2, DN-cDC2, cDC1, lysozyme-expressing DC (LysoDC), plasmacytoid DC (pDC), and lysozyme-expressing macrophage (LysoMac) according to the reported expression patterns of their signature genes (**Figures 4C, D**) (10). Interestingly, DN-cDC2 and LysoMac could be classified into two clusters by the differential expression of marker genes, indicating the functional heterogeneity of these cells (**Figures 4C–E**). All phagocytic cell subsets were decreased in AFD-fed mice except for DN-cDC2_2 (**Figure 4F**). Thus, we next set out to examine food-antigen-regulated functions of DCs that may impact the differentiation of T cells in PPs. We used NicheNet (30) to search for putative ligands expressed by DCs that could act on receptors expressed by naïve T cells and detected 65 ligands that potentially could induce the food-antigen-driven functional changes in naïve T cells (**Figure 5A**). Specifically, not only was *H2-DMa*, a major histocompatibility complex (MHC) gene important for the induction of CD4^+^ T cells, downregulated on cDC2, DN-cDC2, and LysoDC in AFD-fed mice (43), but so too were *H2-M3* and *H2-T23*, MHC genes involved in the induction of CD8^+^ T cells (44, 45) in cDC1, indicating that food antigens themselves regulate the antigen presentation capacity of DCs (**Figures 5B, C**). Supporting this interpretation, we observed changes in the expression of CD40 by cDC2, DN-cDC2, and cDC1 induced by food antigens (**Figures 5B, C**), consistent with a previous report (20).

## Discussion

We here showed that food antigens are important in suppressing tumorigenesis in the small intestine. The novelty of this study lies in that food antigens play a crucial role in suppressing small intestinal tumorigenesis. One could criticize that inflammatory status possibly affects the tumorigenesis in our experiments, but our histological analysis suggested that AFD did not cause any symptoms of the inflammation in our histological analysis (data not shown). A similar effect of AFD on the inflammation has also been shown in the previous report (Kim et al, Science, 2016). Although we could not formally exclude the possibility that BSA and OVA act as additional nutrients rather than as food antigens, it is reasonable to assume that BSA and OVA act as food antigens because the AFD contains enough nutrients in the form of elementary small molecules and the mice apparently live healthy on the AFD. As a mechanism, we propose that the number of CD8^+^ T cells infiltrating the tumors is important for the anti-tumor responses. We speculate that their activity depends on Th1 cells because IFNγ, a cytokine predominantly secreted by Th1 cells, promotes cytotoxicity by CD8^+^ T cells. Indeed, we observed that the number of CD8^+^ T cells in the small intestinal tumors decreased in AFD-fed *APC^min/+^* mice, which is partially restored by the addition of BSA to the AFD. However, our study does not clarify whether such changes depend on IFNγ secreted by Th1 cells induced by food antigens, and this issue remains a subject for future investigation. In our experiments, IgA^+^ plasma cells were decreased in SI LP of AFD-fed mice. Therefore, changes in the microbiota may be involved in the suppression of the small intestinal tumors. However, the changes are not suggested to be involved in the regulation of the small intestinal tumorigenesis because the number of IgA^+^ plasma cells was not restored upon the addition of BSA. A similar phenomenon was observed for Tfh cells, unlike other T cells. The discrepancy between Tfh cells and other T cells could be partly explained by the fact that Tfh cell generation is profoundly limited in adult mice, which is probably a reason why IgA^+^ plasma cells are not restored by the addition of BSA (20). For the induction of IgA, the expression of TGF-β in DCs depends on food antigens, while the expression of IL-21 depends on microbiota (**Supplementary Figures 5A, B**). This suggests that the process relies on the synergistic effects of microbiota and food antigens. Food antigens are taken up into PPs mainly through M cells and delivered to DCs. There, food antigens induce DC maturation by upregulating costimulatory and MHC molecules, probably through enhanced expression of calmodulin. Concerning the antigen specificity in tumor suppression by food antigens, we do not think that it is a crucial factor, since tumor suppression was observed regardless of the kind of food antigens, BSA or OVA. Instead, we propose that antigen-nonspecific immune conditioning/training by food antigens in the small intestine plays an important role in suppressing small intestinal tumorigenesis in the observed *Apc^min/+^* mice. Intestinal ILC1 and ILC2 subsets do not express MHC class II but intestinal LTi-like ILC3 can express MHC class II, which has been described to be involved in controlling adaptive immunity against the intestinal microbiota (46–49). In our scRNA-seq data, the number of PP ILC3 did not change (**Figure 3C**). However, we observed relatively higher expression levels of MHC class I-encoding *H2-T23* gene compared to NK/ILC1 and ILC2 was observed in ILC3 and exILC3, and their *H2-T23* gene expression levels were decreased in AFD-fed mice (**Supplementary Figures 5C, D**). These data suggest the possibility that food antigens can regulate the antigen-presenting function of ILC3. Additionally, food antigens are reported to suppress the proliferation and activation of SI LP ILC2 (22). In our study, we did not observe similar changes in the PP ILC2. This discrepancy between our results and previous reports might be due to differences in the environment housing the AFD-fed mice but further careful research is needed in the future. Regarding DCs, cDC2, DN-cDC2, LysoDC, and LysoMac are considered to induce IgA production, and LysoDC is for Th1 cell activation (9, 10). In our data, the numbers of these DCs were reduced in AFD-fed mice, indicating that the regulation of the number of these DCs by food antigens is involved in the induction of IgA and Th1 cells (**Figure 4F**). Regarding the suppression of tumorigenesis, LysoDC may be involved in the process due to its induction of Th1 cells, which is one of the topics for future study in our research. Additionally, these data suggest the possibility that food antigens could regulate the migration of DCs into the PPs. However, how this migration is regulated is a topic for future investigation. As a limitation, we were unable to detect tingible-body macrophages, which are located in the germinal center of PPs, in our study, presumably due to differences in the methods used for isolating PP cells or the potential mixing with other DC clusters in our scRNA-seq analysis (9, 10). Another point that should be discussed is whether the mesenteric lymph node (MLN) is involved in the phenomenon observed in this study and, if that is true, how the food antigens are delivered to the MLN. In WT mice, we observed that the number of MLN immune cells decreased in AFD-fed mice. However, contrary to the case in PPs, the numbers were not restored by the addition of BSA to the AFD (**Supplementary Figure 6**). These data indicate that the contribution of MLN is minimal or it might require a longer time to observe the role of MLN in this process under our experimental conditions. Supporting this idea, PP-null *Apc* ^min/+^ mice have a similar number of tumors to those of AFD-fed *Apc* ^min/+^ mice (**Figures 1A, F**). However, it is still possible that antigens that once entered PPs are delivered to MLN and contribute to the small intestinal immune responses. At least, bacteria such as *S.* Typhimurium can enter both PPs and MLN, and their entry depends on glycoprotein 2 (GP2), which is specifically expressed on the surface of M cells among enterocytes (38). On the other hand, in case of soluble antigens such as prions, even how they enter PPs is still controversial. One report shows that soluble antigens enter PPs through M cells supporting our results (50), whereas another report shows that soluble antigens enter PPs through FAE cells that are different from M cells (39). Hence, it remains unclear whether soluble or food antigens that have entered PPs can translocate to MLN to induce small intestinal immune cells, and clarification of this mechanism will further elucidate how food antigens suppress tumorigenesis via induction of the immune system in the future. The present findings also have clinical significance. Ingredients of the AFD used in this study resemble those used clinically for enteral nutrition, which is commonly used as support therapy in gastrointestinal malignancies (51). In particular, patients with familial adenomatous polyposis often suffer from small intestinal tumors (52). Enteral nutrition is also a therapeutic option for inflammatory bowel disease (53), which is a well-known tumorigenesis risk factor, including for small intestinal cancer. Thus, careful consideration should be paid prior to application of enteral nutrition in these patients, although more studies in humans will be required before definitive conclusions are reached.

## Data Availability

The data presented in the study are deposited in the NCBI GEO repository, accession number GSE266888.

## Supplementary material

Supplementary Figure 1 Food antigens increase the number of T cells in PPs of GF mice and OVA induces the PP T cells. **(A)** WT mice kept under GF conditions were placed on an NCD, AFD, or 5% BSA-AFD for 4 weeks starting at 4 weeks of age, and the numbers of PP T cells, CD4^+^ T cells, and CD8^+^ T cells, including naive cells, effector cells, and Tfh cells, were determined by flow cytometry analysis (*n* = 4–5). **(B)** WT mice were placed on an AFD or 5% OVA-AFD under SPF conditions for 4 weeks starting at 4 weeks of age, and the numbers of PP T cells, CD4^+^ T cells, and CD8^+^ T cells, including naive cells and effector cells, in PPs were determined by flow cytometry analysis (*n* = 3–5). Data are presented as mean ± SD. Statistical significance was calculated by Tukey–Kramer test **(A)** and Mann–Whitney *U*-test **(B)**. **p* < 0.05 and ***p* < 0.01.

Supplementary Figure 2 Influence of food antigens on the induction of intestinal T cells in WT mice. **(A, B)** WT mice housed under SPF **(A)** or GF **(B)** conditions were placed on an NCD, AFD, or AFD supplemented with 5% BSA for 5 weeks starting at 4 weeks of age, and the numbers of SI LP T cells, CD4^+^ T cells, Th1 cells, Treg cells, CD8^+^ T cells, and IgA^+^ plasma cells were determined by flow cytometry analysis (*n* = 4–5). **(C)** WT mice housed under SPF conditions were placed on an NCD or AFD for 5 weeks starting at 4 weeks of age, and the numbers of LI LP T cells, CD4^+^ T cells, Th1 cells, Treg cells, and CD8^+^ T cells were determined by flow cytometry analysis (*n* = 5). Data are presented as mean ± SD. Statistical significance was calculated by Tukey–Kramer test **(A, B)** and Mann–Whitney *U*-test **(C)**. **p* < 0.05 and ***p* < 0.01.

Supplementary Figure 3 Involvement of M cells in the regulation of small intestinal tumorigenesis. **(A–C)** The number of total small intestinal tumors **(A)**, the numbers of the tumors in the small intestine equally divided into three parts (upper, middle, and lower) (B), and sizes of the tumors **(C)** were measured in *Villin* ^+/+^ *Spib* ^flox/flox^ *Apc* ^min/+^ or *Villin* ^+/+^ *Spib* ^flox/+^ *Apc* ^min/+^ (Control) mice and *Villin* ^Cre/+^ *Spib* ^flox/flox^ (*Spib*ΔIEC) *Apc* ^min/+^ mice (*n* = 3). Data are presented as mean ± SD. Student’s *t*-test.

Supplementary Figure 4 Top 10 marker genes for each lymphocyte population. Expression of the top 10 marker genes for each lymphocyte cluster is illustrated as a heatmap using Seurat.

Supplementary Figure 5 The expression of genes required for IgA production and H2-T23 expression in ILC3 and exILC3. **(A)** Average expression and percentage of Tfh cells expressing Il4 and Il21. **(B)** Average expression and percentage of cells expressing Tnfsf13, Tnfsf13b, and Tghb1 within the indicated DC subsets in SPF and GF mice maintained on an NCD or AFD. **(C)** Average expression and percentage of cells expressing ILC markers for indicated ILC subsets, along with H2-T23 expression within the indicated ILC subsets. **(D)** The expression levels of the H2-23 gene in ILC3 and exILC3 in SPF and GF mice maintained on an NCD or AFD. **(A–D)** These data were derived from our scRNA-seq analysis and represent combined data from 2 mice.

Supplementary Figure 6 Induction of immune cells in the mesenteric lymph node (MLN) by food antigens. WT mice kept under SPF conditions were placed on an NCD, AFD, or 5% BSA-AFD for 4 weeks starting at 4 weeks of age, and the numbers of MLN T cells, CD4^+^ T cells, and CD8^+^ T cells, including naive cells, effector cells, and Tfh cells, were determined by flow cytometry analysis (*n* = 4–5). Data are presented as mean ± SD. Statistical significance was calculated by Tukey–Kramer test. **p* < 0.05 and ***p* < 0.01.

Supplementary Figure 7 Involvement of Peyer’s patches in the induction of SI immune cells in male mice. Related to **Figure 2B** using female mice, data were obtained for PP-null male mice fed NCD/AFD from another experiment, suggesting that similar results were obtained between male and female mice. The numbers of SI LP T cells and plasma cells were determined by flow cytometry analysis (*n* = 6–7). Data are presented as mean ± SD. Statistical significance was calculated by Tukey–Kramer test. **p* < 0.05 and ***p* < 0.01.

Supplementary Figure 8 Similar effects of food antigens on small intestinal tumorigenesis in male and female *Apc^min/+^* mice. **(A)** The data in **Figure 1A** were divided into male and female subsets and presented separately. **(B)** The data in **Figure 1D** were divided into male and female subsets and presented separately.
