# Supplementary figures and images for: Food antigens suppress small intestinal tumorigenesis
Source: Front Immunol. 2024 Sep 18;15:1373766. doi: 10.3389/fimmu.2024.1373766 (PMC11445177; doi:10.3389/fimmu.2024.1373766)

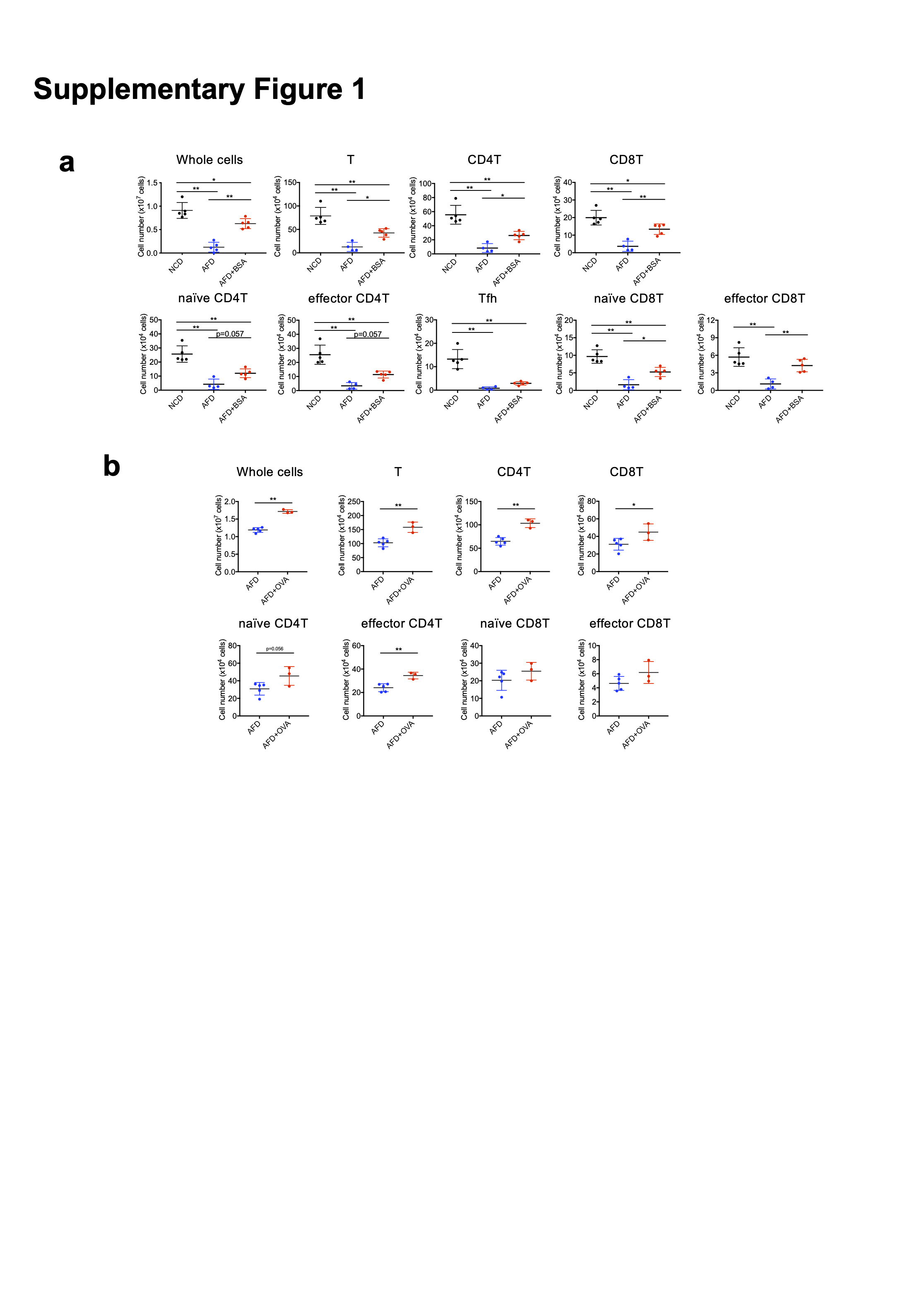

Supplement: Supplementary Figure 1 — Food antigens increase the number of T cells in PPs of GF mice and OVA induces the PP T cells. (A) WT mice kept under GF conditions were placed on an NCD, AFD, or 5% BSA-AFD for 4 weeks starting at 4 weeks of age, and the numbers of PP T cells, CD4+ T cells, and CD8+ T cells, including naive cells, effector cells, and Tfh cells, were determined by flow cytometry analysis (n = 4–5). (B) WT mice were placed on an AFD or 5% OVA-AFD under SPF conditions for 4 weeks starting at 4 weeks of age, and the numbers of PP T cells, CD4+ T cells, and CD8+ T cells, including naive cells and effector cells, in PPs were determined by flow cytometry analysis (n = 3–5). Data are presented as mean ± SD. Statistical significance was calculated by Tukey–Kramer test (A) and Mann–Whitney U-test (B). *p < 0.05 and **p < 0.01. [file Image1.jpg]

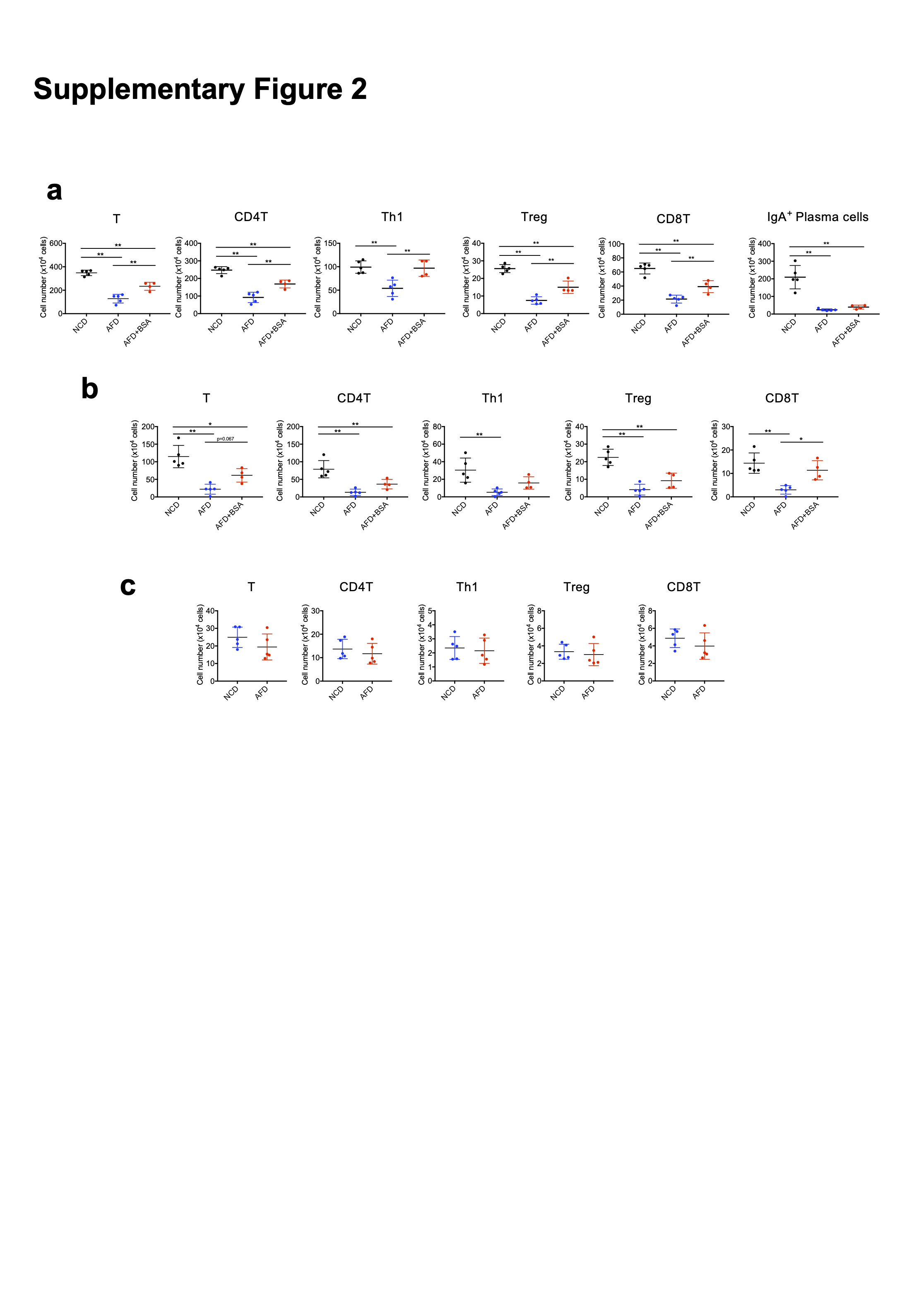

Supplement: Supplementary Figure 2 — Influence of food antigens on the induction of intestinal T cells in WT mice. (A, B) WT mice housed under SPF (A) or GF (B) conditions were placed on an NCD, AFD, or AFD supplemented with 5% BSA for 5 weeks starting at 4 weeks of age, and the numbers of SI LP T cells, CD4+ T cells, Th1 cells, Treg cells, CD8+ T cells, and IgA+ plasma cells were determined by flow cytometry analysis (n = 4–5). (C) WT mice housed under SPF conditions were placed on an NCD or AFD for 5 weeks starting at 4 weeks of age, and the numbers of LI LP T cells, CD4+ T cells, Th1 cells, Treg cells, and CD8+ T cells were determined by flow cytometry analysis (n = 5). Data are presented as mean ± SD. Statistical significance was calculated by Tukey–Kramer test (A, B) and Mann–Whitney U-test (C). *p < 0.05 and **p < 0.01. [file Image2.jpg]

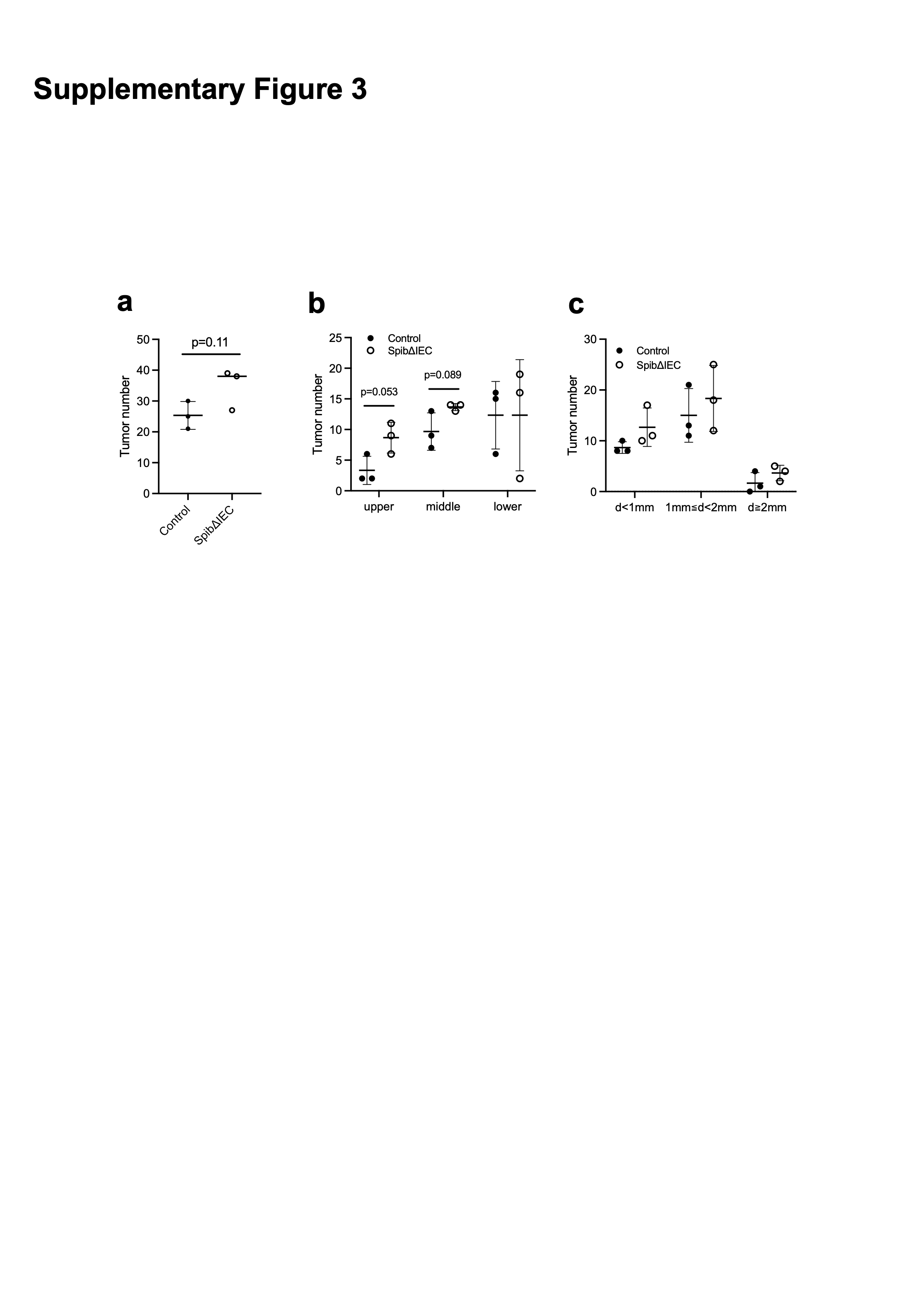

Supplement: Supplementary Figure 3 — Involvement of M cells in the regulation of small intestinal tumorigenesis. (A–C) The number of total small intestinal tumors (A), the numbers of the tumors in the small intestine equally divided into three parts (upper, middle, and lower) (B), and sizes of the tumors (C) were measured in Villin +/+ Spib flox/flox Apc min/+ or Villin +/+ Spib flox/+ Apc min/+ (Control) mice and Villin Cre/+ Spib flox/flox (SpibΔIEC) Apc min/+ mice (n = 3). Data are presented as mean ± SD. Student’s t-test. [file Image3.jpg]

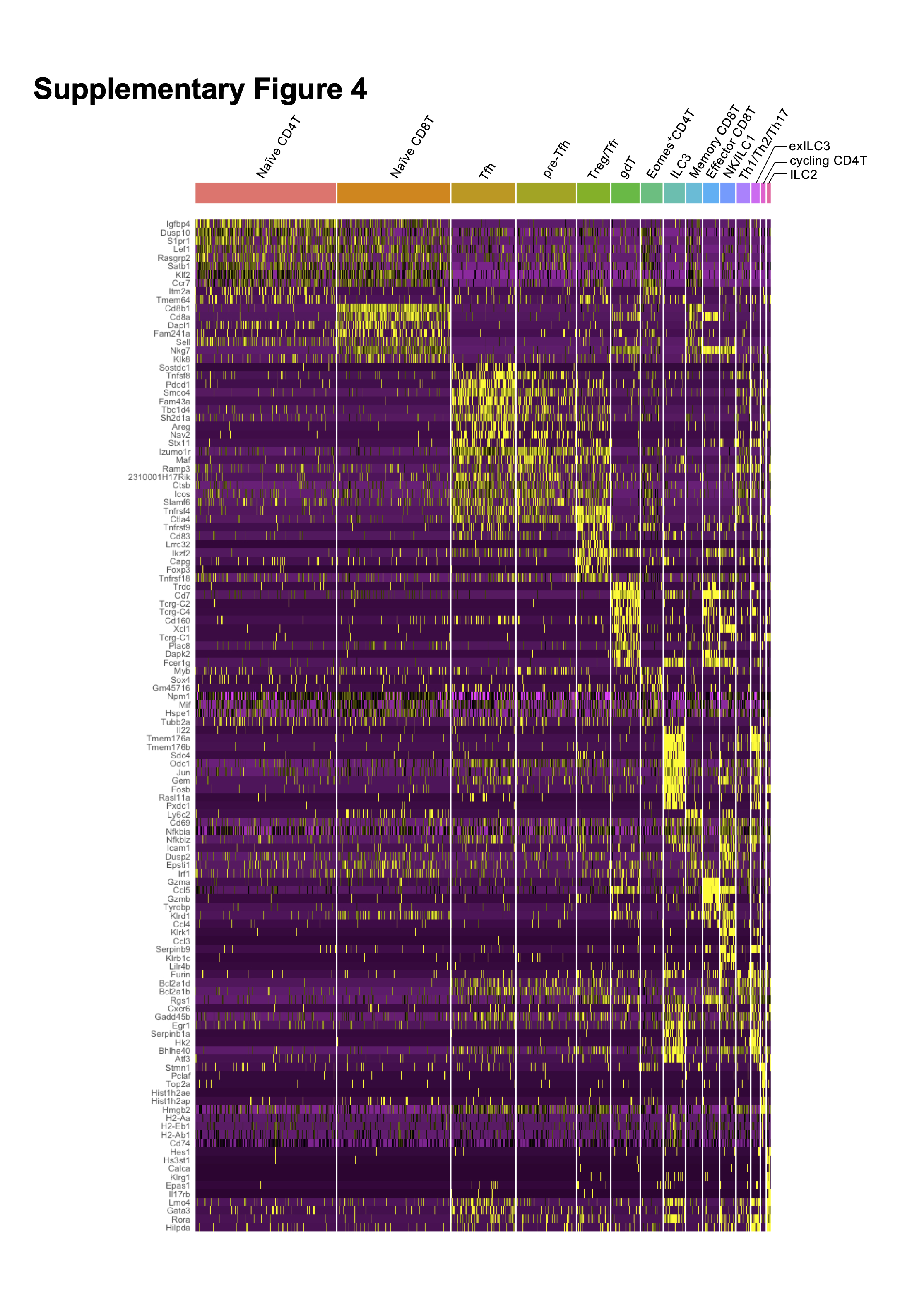

Supplement: Supplementary Figure 4 — Top 10 marker genes for each lymphocyte population. Expression of the top 10 marker genes for each lymphocyte cluster is illustrated as a heatmap using Seurat. [file Image4.jpg]

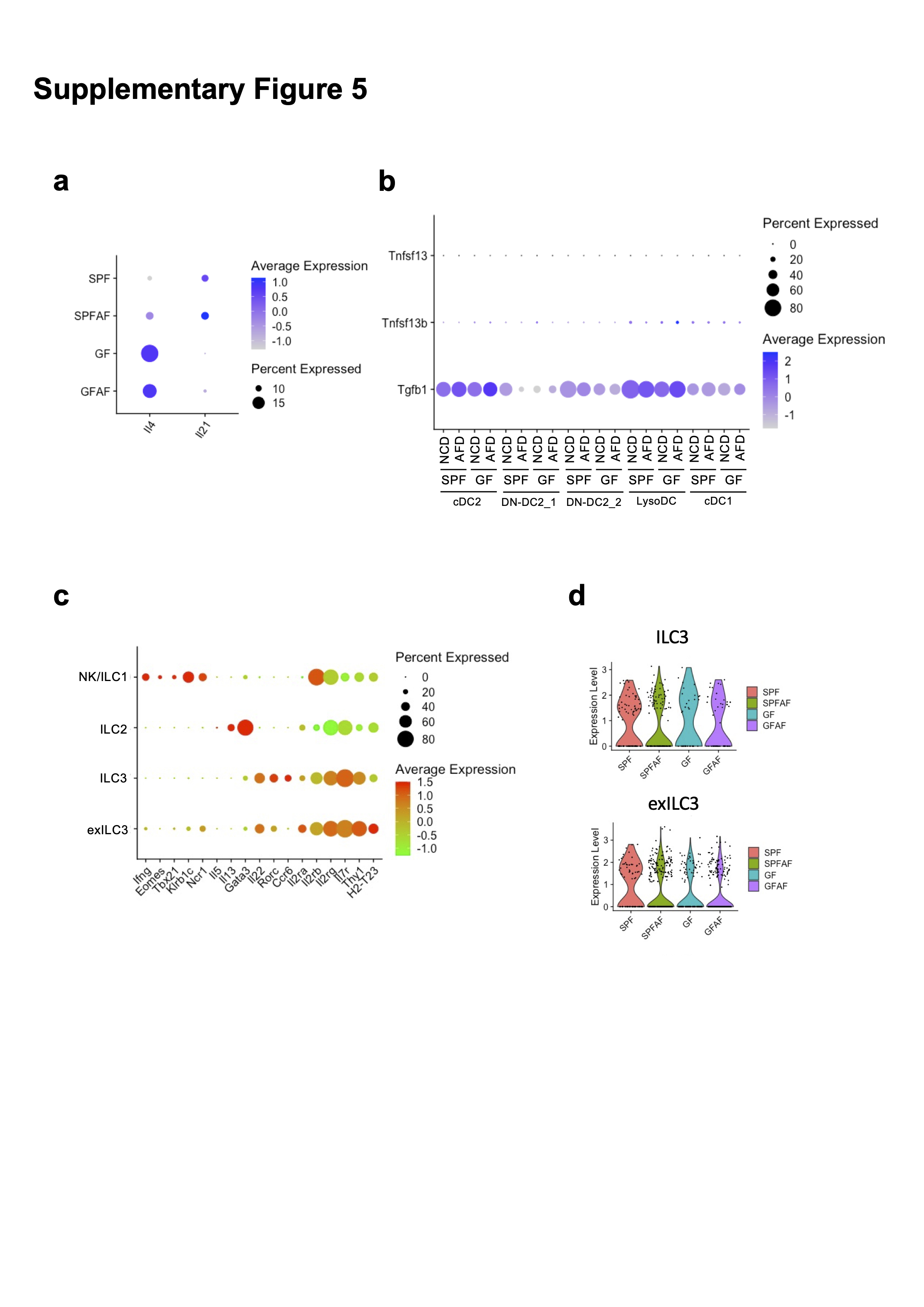

Supplement: Supplementary Figure 5 — The expression of genes required for IgA production and H2-T23 expression in ILC3 and exILC3. (A) Average expression and percentage of Tfh cells expressing Il4 and Il21. (B) Average expression and percentage of cells expressing Tnfsf13, Tnfsf13b, and Tghb1 within the indicated DC subsets in SPF and GF mice maintained on an NCD or AFD. (C) Average expression and percentage of cells expressing ILC markers for indicated ILC subsets, along with H2-T23 expression within the indicated ILC subsets. (D) The expression levels of the H2-23 gene in ILC3 and exILC3 in SPF and GF mice maintained on an NCD or AFD. (A–D) These data were derived from our scRNA-seq analysis and represent combined data from 2 mice. [file Image5.jpg]

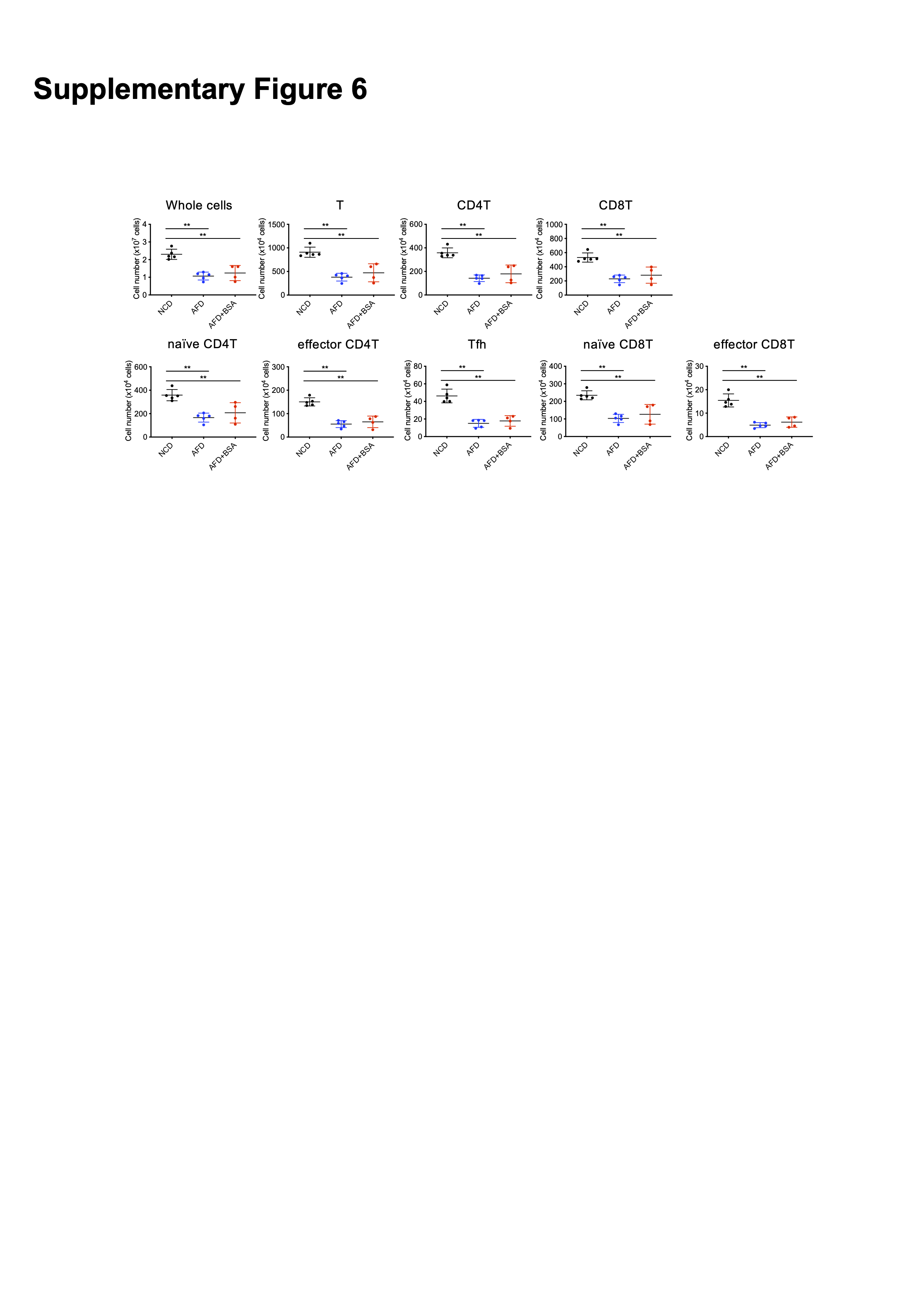

Supplement: Supplementary Figure 6 — Induction of immune cells in the mesenteric lymph node (MLN) by food antigens. WT mice kept under SPF conditions were placed on an NCD, AFD, or 5% BSA-AFD for 4 weeks starting at 4 weeks of age, and the numbers of MLN T cells, CD4+ T cells, and CD8+ T cells, including naive cells, effector cells, and Tfh cells, were determined by flow cytometry analysis (n = 4–5). Data are presented as mean ± SD. Statistical significance was calculated by Tukey–Kramer test. *p < 0.05 and **p < 0.01. [file Image6.jpg]

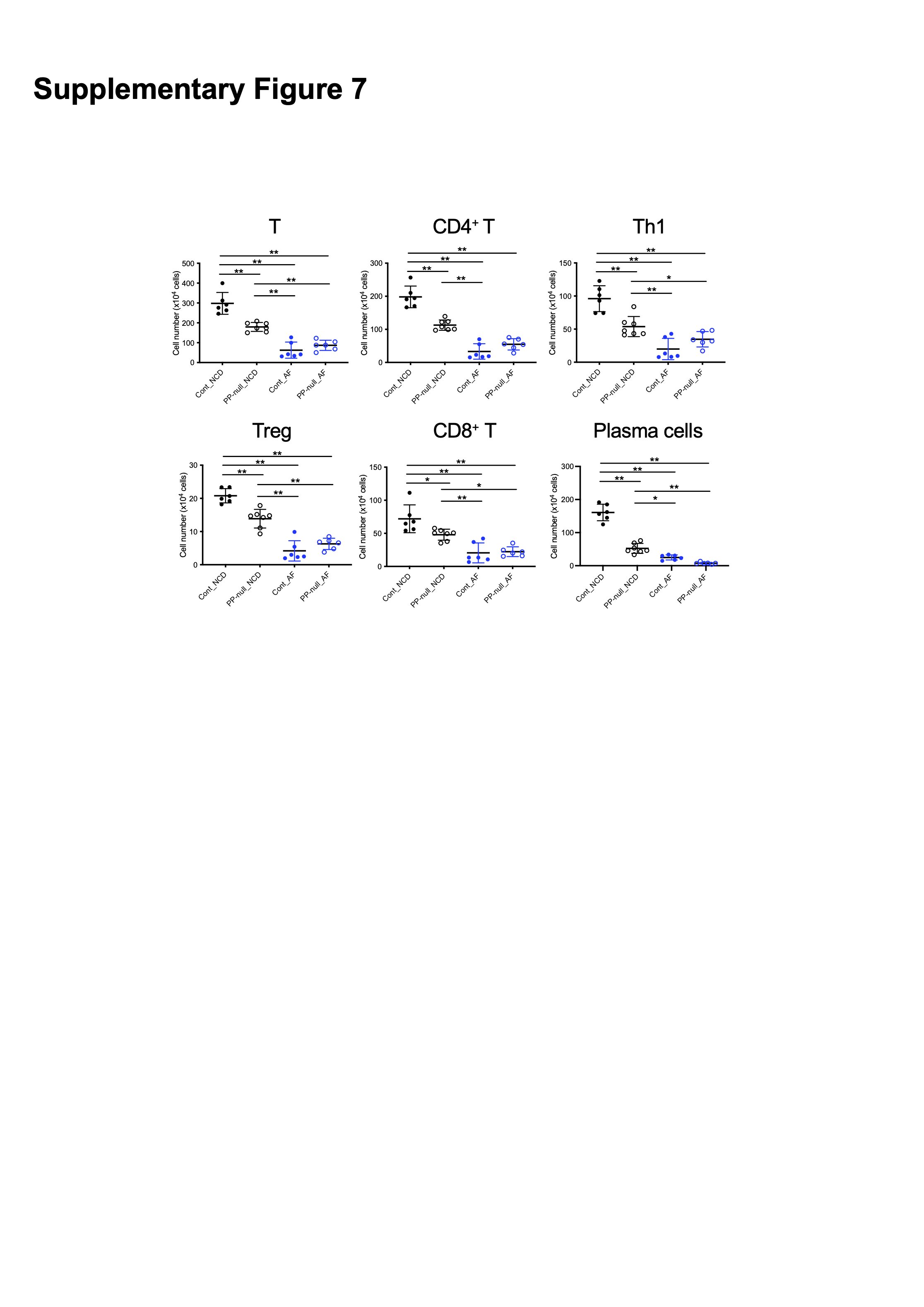

Supplement: Supplementary Figure 7 — Involvement of Peyer’s patches in the induction of SI immune cells in male mice. Related to Figure 2B using female mice, data were obtained for PP-null male mice fed NCD/AFD from another experiment, suggesting that similar results were obtained between male and female mice. The numbers of SI LP T cells and plasma cells were determined by flow cytometry analysis (n = 6–7). Data are presented as mean ± SD. Statistical significance was calculated by Tukey–Kramer test. *p < 0.05 and **p < 0.01. [file Image7.jpg]

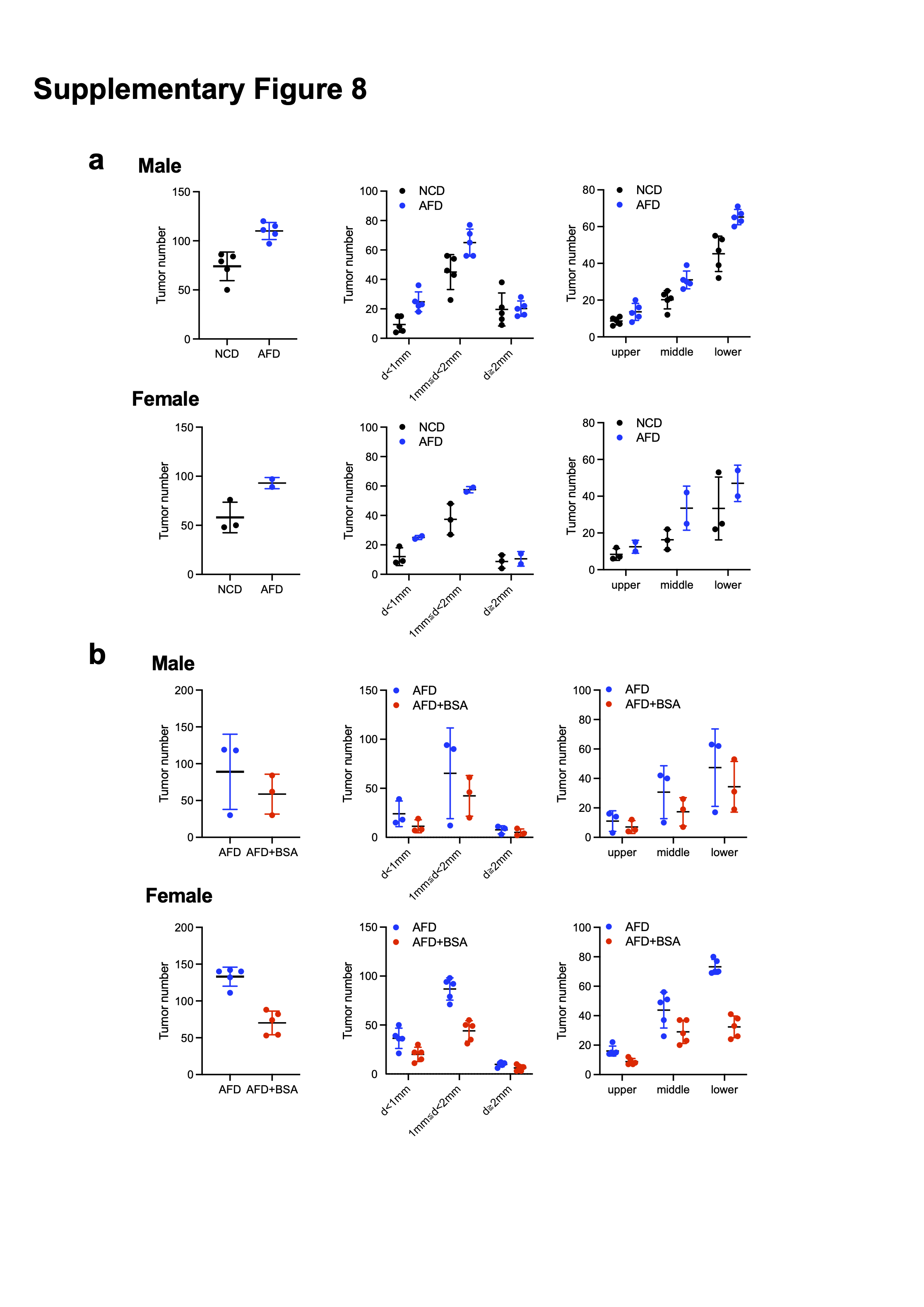

Supplement: Supplementary Figure 8 — Similar effects of food antigens on small intestinal tumorigenesis in male and female Apcmin/+ mice. (A) The data in Figure 1A were divided into male and female subsets and presented separately. (B) The data in Figure 1D were divided into male and female subsets and presented separately. [file Image8.jpg]
